# Drug Repurposing for Rare Diseases: A Role for Academia

**DOI:** 10.3389/fphar.2021.746987

**Published:** 2021-10-20

**Authors:** Sibren van den Berg, Saco de Visser, Hubert G.M. Leufkens, Carla E.M. Hollak

**Affiliations:** ^1^ Medicine for Society, Platform at Amsterdam UMC—University of Amsterdam, Amsterdam, Netherlands; ^2^ Department of Endocrinology and Metabolism, Amsterdam UMC—University of Amsterdam, Amsterdam, Netherlands; ^3^ Division of Pharmacoepidemiology and Clinical Pharmacology, Utrecht Institute for Pharmaceutical Sciences (UIPS), Utrecht University, Utrecht, Netherlands

**Keywords:** drug repurposing, mexiletine, etidronate, abatacept, orphan drugs, off-label, reimbursement, rare diseases

## Abstract

**Background:** The European Commission highlights in its Pharmaceutical Strategy the role of academic researchers in drug repurposing, especially in the development of orphan medicinal products (OMPs). This study summarizes the contribution of academia over the last 5 years to registered repurposed OMPs and describes barriers to success, based upon three real world cases.

**Methods:** OMPs granted marketing authorization between January 2016 and December 2020 were reviewed for repurposing and whether the idea originated from academia or industry. Three cases of drug repurposing were selected from different therapeutic areas and stages of development to identify obstacles to success.

**Results:** Thirteen of the 68 OMPs were the result of drug repurposing. In three OMPs, there were two developments such as both a new indication and a modified application. In total, twelve developments originated from academia and four from industry. The three cases showed as barriers to success: lack of outlook for sufficient return of investments (abatacept), lack of regulatory alignment and timing of interaction between healthcare professionals and regulators (etidronate), failure to register an old drug for a fair price, resulting in commercialization as a high priced orphan drug (mexiletine).

**Conclusion:** While the majority of repurposed OMPs originates in academia, a gap exists between healthcare professionals, regulators and industry. Future strategies should aim to overcome these hurdles leading to more patient benefit through sustainable access of repurposed drugs. Potential solutions include improved regulatory and reimbursement knowledge by academia and the right for regulators to integrate new effectiveness data into product labels.

## Introduction

Drug repurposing, or drug repositioning, is the application of an already known active substance in a new way—such as a new indication or alternative method of presentation (Langedijk et al., 2015). The major advantage of drug repurposing is the availability of clinical and regulatory knowledge on the active substance’s safety profile, pharmacokinetics, dose, quality and production process, hence typically lowering overall risk and development costs (Sardana et al., 2011; Pushpakom et al., 2018). Drug repurposing has especially been coined as a possible relevant strategy for development of medicines for rare diseases (Caban et al., 2017; Davies et al., 2017; Tambuyzer et al., 2020; Kort & Jovinge., 2021). A recent analysis showed that almost half of the drug repurposing collaborations in the Excelra database were targeted at rare diseases (Polamreddy and Gattu, 2019). However, drug repurposing often does not lead to formal regulatory approval due to a variety of legal, regulatory and market constraints, among others. First, since newly discovered treatment targets are frequently reported in literature, it may be difficult or impossible to obtain intellectual property protection. In addition, the strength of second-use patents to protect against competitors is often weak (Pushpakom et al., 2018; Verbaanderd et al., 2020). Second, additional costly clinical development investments may be needed to prove efficacy for the new indication, as well as possible additional requirements with respect to dosing and safety (Kort & Jovinge., 2021; Verbaanderd et al., 2021). These additional costs may limit the prospects for sufficient profitability, especially when low-priced generic versions of the originator are already used off-label (Breckenridge & Jacob., 2018; Verbaanderd et al., 2020). Off-label use may by itself be problematic: if the level of evidence for the new application is low, access, pharmacovigilance and reimbursement may be variable.

In November 2020, the European Commission highlighted in its Pharmaceutical Strategy for Europe the role of academic researchers and not-for-profit stakeholders to promote and develop repurposing of off-patent medicines for new therapeutic uses (European Commission, 2020). Industry engagement as part of public-private partnerships in this process is emphasized to close the loop to formal authorisation, as industry has valuable experience and knowledge about regulatory processes. However, academia may face several obstacles to successfully operate in this field: for example a lack of infrastructure, resources and expertise in regulatory affairs and academic incentives for fast publications, hampering the protection of intellectual property (Verbaanderd et al., 2020).

Because drug repurposing has the potential to deliver treatments to patients with rare diseases with an unmet medical need, it is important that such discoveries also become available and accessible for patients. In this exploratory study, we therefore addressed the following research questions: 1) What is the contribution of academia over the last 5 years regarding authorised drug repurposing for rare diseases? 2) What are the hurdles that hinder drug repurposing for rare diseases started by academia? We answer these questions by looking at the origin of drug repurposing of authorised orphan medicinal products (OMPs) and by describing three real world ongoing cases of drug repurposing by academia for rare diseases in different stages of development.

### Methods

To determine the contribution of academia to drug repurposing for rare diseases, we selected all OMPs with a valid marketing authorization granted by the European Commission between January 2016 and December 2020 (68 OMPs). Data extraction was performed on December 16th, 2020 from the EMA website (EMA, 2020).

A drug was defined as “repurposed” when the active substance was either used in clinical practice for another indication, or for the same indication, but with a modified application (e.g., other formulation/mode of administration). The original indication or application should have been in place for at least 10 years before the marketing authorization of the OMP to exclude new active substances. For each OMP, PubMed was searched to retrieve published evidence of prior clinical use and analyzed whether the active substance was registered for the original indication 1) and/or for another indication 2) (“Indication”). If 1) was the case, we investigated whether there was a modified application (“Modified”). The results were grouped by anatomical therapeutic chemical classification system (ATC) code, that classifies active ingredients based on anatomic, therapeutic and pharmacologic properties (WHO Collaborating Centre for Drug Statistics Methodology, 2021).

Whether the drug repurposing originated from academia or industry was determined upon the first description of the development (Indication or Modified application) in scientific publications (PubMed) or clinicialtrials.gov. Affiliations, sponsors, acknowledgements and conflict of interest (CoI) statement were reviewed. If there was at least one commercial entity involved in one of those domains, the development was labelled as initiated by industry. If there were only academic entities involved or when the full text described the emergence of the idea in academia, the development was labelled as initiated by academia.

To identify and elaborate on hurdles for drug repurposing for rare diseases whose development starts in academia, we purposively selected three cases to show the diversity and variation of issues that hinder drug repurposing for rare diseases from academia. All cases take place in the Netherlands and came to our attention through national media or through activities for the academic platform “Medicine for Society” (www.medicijnvoordemaatschappij.nl) (Volkskrant, 2018; Volkskrant, 2019). One author (SvdB) held unstructured interviews with the involved researchers from academic medical centers, who are all physicians treating patients with the rare disease. Afterwards, the interviewees verified the findings and gave consent for publication. As the study does not fall under the definition used for medical scientific research, it has therefore not been assessed by the medical ethics committee. The three selected real life cases represent different therapeutic areas and stage of development: a case in the area of immunology, in early developmental stage with only a few published case reports, a case in the area of metabolism where clinical trials have been performed and a case in the area of neurology where an old drug was registered as an OMP.

## Results

### Contribution of Academia to Drug Repurposing for Rare Diseases

**FIGURE 1:**
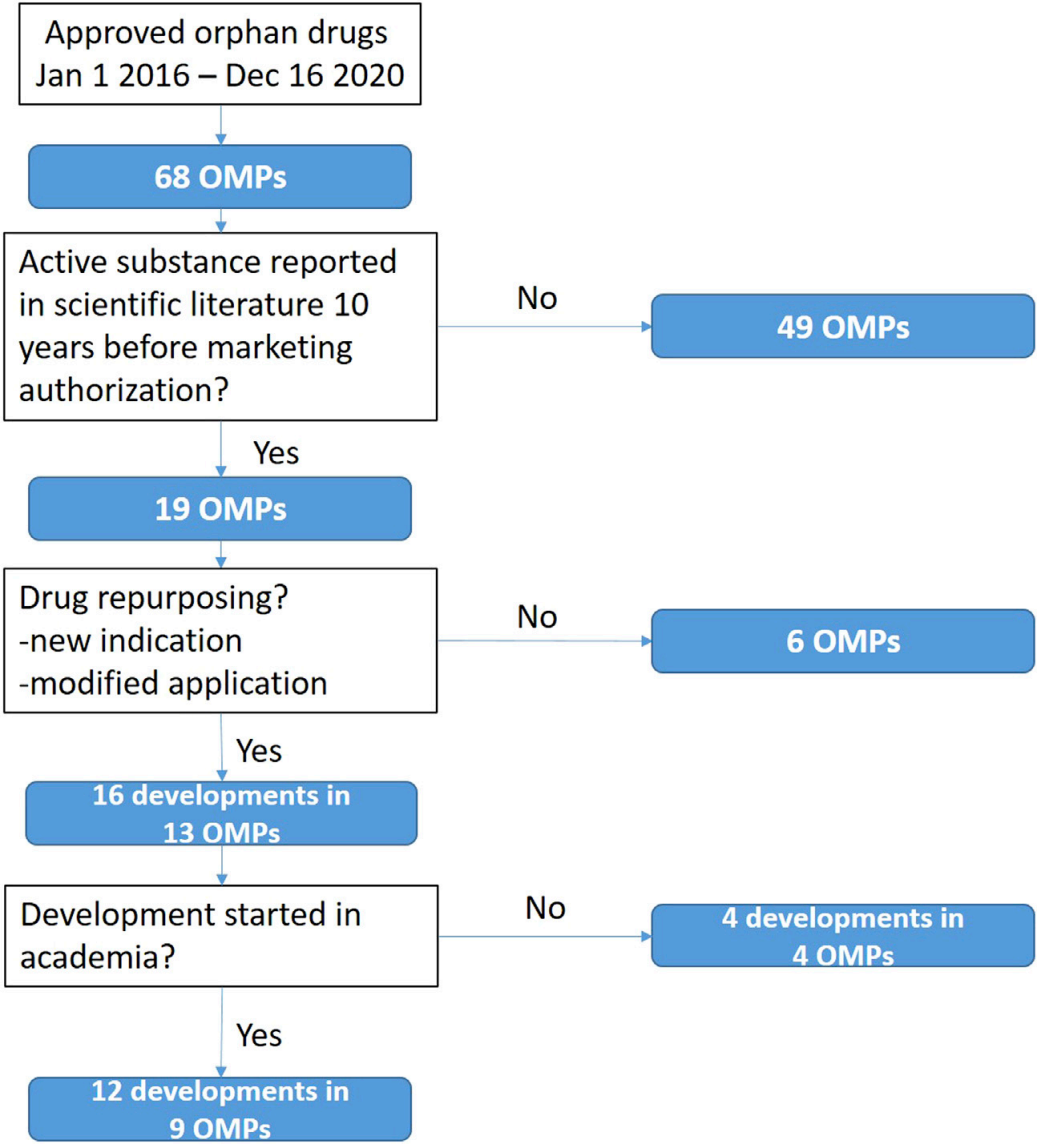
Outline of the process to identify repurposed orphan medicinal products approved by the European Medicines Agency.

Thirteen of 68 OMPs licensed in Europe during a 5-year period (2016–2020) were repurposed drugs (Figure 1). Three OMPs have been repurposed twice (e.g., both indication and formulation), leading to 16 developments. Twelve developments (75%) in nine OMPs started in academia and four developments (25%) in four OMPs started in industry. Ten of the 12 (83%) academia-originating developments were for a new therapeutic indication, while 75% (3/4) developments started in industry were a modified application. Table 1 presents an overview of all repurposed orphan drugs and the nature of the developments.

**TABLE 1:** Repurposed orphan medicinal products approved between January 1st, 2016 and December 16th, 2020. CoI: conflict of interest.

Active substance	Therapeutic indication (current therapeutic indication in case of only a “Modified” innovation, otherwise previous therapeutic indication)	Innovation	Trade name	Pharmaceutical form	Start of drug repurposing	ATC Class current indication	References	Remarks
Amikacin sulfate	Infections with Gram-negative and Gram-positive organisms	Indication: Non-tuberculous mycobacterial lung infections	Arikayce liposomal	Nebuliser dispersion	Indication: Academia	J—Antiinfectives for systemic use	Forslöw et al. (2003)	No CoI, acknowledgement suggests no industry funding
		Modified: Liposomal			Modified: Industry		Weers et al. (2009)	
Budesonide	Asthma, allergic rhinitis, nasal polyps	Indication: Eosinophilic esophagitis	Jorveza	Orodispersible tablet	Academia	A—Alimentary tract and metabolism	Aceves et al. (2007)	No acknowledgement, CoI describes industry funding
Cannabidiol	Lennox Gastaut syndrome, Dravet syndrome, seizures associated with tuberous sclerosis complex	Indication: Registered for subset of known indications	Epidyolex	Oral solution	Academia	N—Nervous system	Izquierdo et al. (1973)	No CoI, acknowledgement suggests no industry funding
Chenodeoxy-cholic acid	Gallstones	Indication: Cerebrotendinous xanthomatosis	Chenodeoxy-cholic acid Leadiant	Hard capsule	Academia	A—Alimentary tract and metabolism	Salen et al. (1974)	No CoI, acknowledgement suggests no industry funding
Chlormethine	Mycosis fungoides-type cutaneous T-cell lymphoma	Modified: Gel	Ledaga	Gel	Industry	L—Antineoplastic and immunomodulating agents	Lessin et al. (2013)	
Ciclosporin	Prevention of graft rejection following solid organ transplantation	Indication: Severe vernal keratoconjunctivitis	Verkazia	Eye drops	Academia	S—Sensory organs	BenEzra et al. (1986)	No full text, no CoI, no acknowledgement
Daunorubicin hydrochloride, cytarabine	Newly diagnosed, therapy-related acute myeloid leukemia or acute myeloid leukemia with myelodysplasia-related changes	Modified: Combination therapy	Vyxeos liposomal	Powder for concentrate for solution for infusion	Modified: Academia	L—Antineoplastic and immunomodulating agents	Crowther et al. (1970)	No CoI, acknowledgement mentions industry but introduction describes the choice of compounds from academia
		Modified: Combination as liposomal			Modified: Industry		Tardi et al. (2009)	
Glibenclamide	Diabetes mellitus type 2	Indication: Neonatal diabetes mellitus	Amglidia	Oral suspension	Academia	A—Alimentary tract and metabolism	Sagen et al. (2004); Zung et al. (2004)	Both: no CoI, acknowledgement describes government funding
Irinotecan hydrochloride trihydrate	Colorectal cancer, small cell lung cancer	Indication: Metastatic adenocarcinoma of the pancreas	Onivyde pegylated liposomal	Concentrate for dispersion for infusion	Academia	L—Antineoplastic and immunomodulating agents	Sakata et al. (1994); Wagener et al. (1995)	Sakata: no full text, no CoI, no acknowledgement. Wagener: No CoI, no acknowledgement
Mercaptamine hydrochloride	Cystinosis	Indication: Corneal cystine crystal deposits	Cystadrops	Eye drops solution	Indication: Academia	S—Sensory organs	Dufier et al. (1987)	No full text, no CoI, no acknowledgement
		Modified: viscous formulation			Modified: Academia		Bozdag et al. (2008)	No CoI, acknowledgement suggests no industry funding
Mexiletine hydrochloride	Ventricular arrhythmias	Incidation: Non-dystrophic myotonic disorders	Namuscla	Hard capsule	Academia	C—Cardiovascular system	Pouget & Serratrice, (1983)	No full text, no CoI, no acknowledgement
Treosulfan	Ovarian cancer	Indication: Conditioning treatment prior to allogeneic haematopoietic stem cell transplantation	Trecondi	Powder for solution for infusion	Industry	L—Antineoplastic and immunomodulating agents	Schmidt-Hieber, (2007)	
Treprostinil sodium	Pulmonary arterior hypertension	Indication: WHO functional class III or IV and chronic thromboembolic pulmonary hypertension	Trepulmix	Solution for infusion	Academia	B- Blood and blood forming organs	Skoro-Sajer et al. (2007)	No acknowledgement, CoI describes government funding

Most developments in repurposed OMPs are in ATC class L (5, Antineoplastic and immunomodulating agents), followed by ATC class A (3, Alimentary tract and metabolism), S (3, Sensory organs) and J (2, Anti-infectives for systemic use). Other ATC classes appear once. The majority (75%) of the innovations started in industry were in ATC class L.

### Hurdles for Drug Repurposing for Rare Diseases: Three Cases

#### Abatacept for Cytotoxic T-Lymphocyte Antigen 4 Haploinsufficiency: Too Rare for Investment in Trials

Abatacept is a product marketed as Orencia® and available as a subcutaneous injection. It was registered in Europe in 2007 to treat rheumatoid arthritis and a number of other forms of arthritis. Abatacept is an analogue of cytotoxic T-lymphocyte antigen 4 (CTLA-4) that acts as a barrier to T-cell activation and is an important immune modulator. Since a number of years, abatacept is also used off-label to treat patients with the very rare disease CTLA-4 haploinsufficiency (prevalence <1 in 1,000,000), which causes severe immune dysregulation (Kuehn et al., 2014; Lapides & McDonald, 2020). Thus far, case reports of patients treated with abatacept reported a prompt response that resolved the inflammatory condition and substantial clinical improvements (Shields et al., 2016; Lee et al., 2016; Van Leeuwen et al., 2018; Lanz et al., 2021).

The new indication for abatacept as treatment of CTLA-4 haploinsufficiency was discovered in academia and was not included in the license by the original marketing authorization holder. The patent on the formulation of abatacept, disputed but not revoked (Holman, 2019), will expire in 2027 (US8476239B2). In the absence of involvement of the marketing authorization holder in a pivotal clinical trial meeting regulatory standards, it is very unlikely that registration for this rare indication will still occur during the patented period. And after patent expiration, the availability of a cheaper biosimilar would probably hinder a higher price, needed to recoup investments in clinical trials and registration procedures.

Reimbursement of off-label abatacept use will then only be possible on a case-by-case basis, subject to agreements with either the hospital or the individual health insurance company. This situation hampers access to patients, while there is consensus amongst doctors to use abatacept off-label and the rationale for its use based upon its pathophysiological mechanisms is clear. The costs of chronic abatacept treatment differ between patients depending on dosing, but are expected to be above €4,000 per month based on 125 mg every 2 weeks (Zorginstituut Nederland, 2021a). An alternative for national or official reimbursement is acceptance of the treatment by insurance companies as standard of care, after scrutinizing the evidence of effectiveness and safety. In the case of abatacept for CTLA-4 deficiency it may be almost impossible to collect sufficient evidence as there are only few case studies published yet and a small single-center clinical trial (funded by the National Institute of Allergy and Infectious Diseases) is not expected to finish until 2026 (Shields et al., 2016; Lee et al., 2016; Van Leeuwen et al., 2018; NCT03733067). Also, obtaining reimbursement in this case is challenging, time-consuming and procedures differ from country to country. For example in Australia, reimbursement of treatment with abatacept is not available even when genetic sequencing indicates suitability for abatacept treatment (Siggs et al., 2019). An analysis in Germany showed a success rate of 75% of acceptance for reimbursements requests for off-label dermatological indications (Seidenschnur et al., 2017). In Belgium, there is no option to get official reimbursement for off-label use, but occasionally costs are covered by the company or solidarity funds (Dooms et al., 2016).

#### Etidronate for Pseudoxanthoma Elasticum: No Longer Commercially Available

Etidronate is a bisphosphonate and was originally developed to prevent and treat osteoporosis. The product has been replaced by alternative bisphosphonates with a better benefit-risk profile over time (Wiesner et al., 2021). This led to the discontinuation of marketing of virtually all etidronate products in Europe (Ministerie van Volksgezondheid, Welzijn en Sport, 2018). However, drug repurposing experiments in an academic hospital had pointed towards etidronate as the bisphosphonate with the highest potential to delay ectopic mineralization given its predominant inhibition of calcium precipitation and hydroxyapatite binding instead of inhibiting osteoclasts like newer bisphosphonates do (Kranenburg et al., 2018; Bartstra et al., 2020). This investigator-initiated single-center, randomized, placebo-controlled trial with 74 patients held in 2015–2016 showed promising effects of etidronate in patients with pseudoxanthoma elasticum (PXE) (Kranenburg et al., 2018; Bartstra et al., 2020). Hence, the compound could be effective for the treatment of PXE, a rare autosomal recessive disorder (prevalence 1-9 in 100,000) (Orphanet, 2021) that leads to ectopic calcification of elastic tissues, including the arteries, skin and Bruch’s membrane (BM) in the retina. (Bartstra et al., 2020; Risseeuw et al., 2020).

The availability of etidronate has been driven essentially by the dynamics of the osteoporosis market: when better or safer alternatives entered the market, they replaced etidronate. The lack of commercially available etidronate hampered both clinical development and clinical use for the new rare indication by academics. The earlier trial was not designed for regulatory purposes and regulators may need different or additional data for the next steps to commercialization. For example, regulators may prefer other endpoints than clinical researchers or require extensive or long-term safety data. The clinician from the academic hospital who performed the trial was driven by scientific curiosity and the need to treat patients. A lack of understanding of regulatory requirements by clinicians and appropriate timing of interaction with regulators or guidance delayed the commercialization of this academic invention.

#### Mexiletine for Non-dystrophic Myotonia: Failure to Register an Old Drug for a Fair Price

Mexiletine is a class 1b anti-arrhythmic drug and has been on the European market since the 1970s. (Postema et al., 2020). Newer anti-arrhythmic drugs have largely replaced mexiletine, but a small group of patients have no alternative. Over time, mexiletine products have been taken off the European market and since 2004 patient access is maintained by import mainly from Japan, Canada and the United States and local pharmacy preparations (compounding). Next to its use for cardiological indications, mexiletine has been used off-label since the 1980s worldwide for the treatment of non-dystrophic myotonias (NDMs) (Pouget & Serratrice, 1983; Trip et al., 2006). NDMs are rare muscle hyperexcitability disorders and characterized by delayed muscle relaxation after voluntary contraction. This leads to symptoms of pain, fatigue, muscle stiffness and weakness (Stunnenberg et al., 2020). In December 2018, mexiletine received a European marketing authorization as a repurposed OMP for the treatment of NDM. Because it is registered as an OMP, a market exclusivity of at least 10 years apply creating a *de facto* monopoly. The price of the newly registered mexiletine, in the same dosage and method of administration (capsule for oral use), has been criticized heavily and rejected by some payers (Postema et al., 2020; Zorginstituut Nederland, 2021b; National Institute for Care and Health Excellence, 2021).

The orphan drug license of mexiletine for NDM is largely based on academic clinical studies and could be seen as a successful repurposing trajectory because it resulted in an officially registered OMP. However, the unexpected price increase has had an opposite effect, hampering access for both indications instead of stimulating rare disease treatment accessibility.

## Discussion

The data indicate that in recent years about one out of five OMPs has been repurposed. This is similar to findings by Davies et al. (2017). Langedijk et al. (2016) found, not specific for OMPs, that 13% of all approved drugs by EMA in 2014 and 2015 were repurposed. We established that developments that start in academia encompass mainly the advancement of existing drugs for new indications in a diverse set of therapeutic areas. In contrast, the developments that started in industry mostly focus on modified applications and the field of oncology. This stresses the potential of academia driven drug repurposing to benefit a broader range of patients.

However, the three cases illustrate that academia faces a diversity of hurdles in different stages of drug development. We summarized the issues by key actor involved as outlined in Table 2. The main hurdles from the side of healthcare professionals involved in drug repurposing were that they have little knowledge about regulatory and reimbursement processes and instead are focused on scientific progress and patient care. In the case of etidronate for pseudoxanthoma elasticum for example, clinicians seem to focus mainly on providing the scientific evidence in drug repurposing and due to lack of knowledge about the regulatory framework as well as restricted time in academia, ideas for drug repurposing might fail unnecessarily or are prematurely halted (Verbaanderd et al., 2020; Starokozhko et al., 2021). In addition, when a drug is available for patients and reimbursed without registration, e.g., as off-label use, they may not be motivated and incentivized to assist in steps towards commercialization. This is supported by the findings of Dooms et al. (2016). Another scenario is that they fail to create access to patients, since they are not aware that regulatory authorities and payers keep other – often higher – standards for either registration or reimbursement. Similar issues were identified for abatacept for CTLA-4 haploinsufficiency. This case, however, in addition highlights, the perceived lack of perspective of sufficient return of investments. The very small and uncertain market in combination with a product already being available for another indication may de-incentivize commercial development and reimbursement. A similar situation may have existed for mexiletine. This drug was ultimately marketed as a repurposed orphan drug for an extremely high price. No private party or academic initiative had led to a timely intervention, to secure access to patients through a formal authorization procedure. Also in this case, without the incentive of market exclusivity, investors are probably reluctant to go through the burden of compiling a dossier. However, monopolization of the market to re-introduce old drugs–as also was the case for CDCA (Sheldon, 2018)—should not be encouraged (Postema et al., 2020). In fact, the orphan drug regulation has never been set up to stimulate this kind of developments, which may even have the opposite effect: drugs become inaccessible due to the extreme price (Technopolis Group & Ecorys, 2020). This, and also the length of market exclusivity has received attention in the evaluation of the orphan drug legislation which is currently taking place (European Commission, 2021). The outcomes of the evaluation could impact drug repurposing for rare diseases as specific incentives, such as the market exclusivity, may change.

**TABLE 2:** Identified hurdles in the three cases of drug repurposing for rare diseases. CTLA-4 HIS: cytotoxic T-lymphocyte antigen four haploinsufficiency. PXE: pseudoxanthoma elasticum. NDM: Non-dystrophic myotonia.

	Hurdles	Abatacept CTLA-4 HIS	**Etidronate PXE**	**Mexiletine NDM**
Healthcare professionals involved in drug repurposing	1. Lack of knowledge of and alignment with the regulatory and reimbursement frameworks	Obtaining reimbursement is challenging and time-consuming	No incentive for clinicians to engage in regulatory activities	—
	2. Off-label use is not a major concern if sufficiently supported by scientific evidence. This slows down evidence development	Consensus amongst doctors based upon pathophysiological mechanisms	Unavailability hampers clinical use and scientific development	Has been used off-label since the 1980s
Private sector	3. Private actors do not invest because of uncertain regulatory and reimbursement outcomes	Viable business case not likely due to patent expiration	—	—
	4. Failure to register an old drug for a fair price, resulting in commercialization as a high priced orphan drug	—	—	Price increase as a result of monopoly position
Payers	5. Hesitant to pay for off-label use, when scientific evidence is limited	Reimbursement only on case-by-case basis	—	—
Regulators	6. Regulatory frameworks not fully adapted for repurposing, both in terms of processes and data/evidence requirements	Case studies not eligible for regulatory purposes	Investigator-initiated trial not eligible for regulatory purposes	—
	7. Regulators are not used to other types of applicants than industry, e.g. academics, doctors, public-private partnerships	—	No appropriate guidance or timing of interaction	—

### Limitations

The cases illustrate some hurdles but we certainly did not provide a structured review of all potential barriers to development of repurposed orphan drugs.

The 5-year period 2016–2020 that we investigated is the most recent period but not necessarily a good reflection of the dynamics in the OMP market. The regulation on OMPs in the European Union went into force in 2000 and until 2017, 142 OMPs were authorized (Technopolis Group & Ecorys, 2020). More than half of these OMPs were authorized between 2012 and 2017. Also, the therapeutic areas of authorized OMPs shifted over time (Technopolis Group & Ecorys, 2020). Altogether, the nature of OMPs, and also the amount of drug repurposing, may have differed in more distant time periods. Although we have shown that indeed drug repurposing by academia plays an important role for OMPs, it would also be interesting to study their contribution to development of non-orphans as a comparison.

In addition, industry may have been involved in more drug repurposing activities than we were able to trace due to publication bias or the availability of only brief abstracts. Also, for older publications the CoI statements were sometimes not included in an article where collaborations with industry may have otherwise been mentioned. Lastly, although we show that drug repurposing for rare diseases mostly starts in academia, it is unclear how the contribution of academia relates to the contribution of industry in registering an OMP. It would be interesting to investigate what activities still had to be done from the moment that industry got involved. This may help to smoothen the collaboration between academia and industry.

### Recommendations and Outlook to the Future

An integrated solution for the described hurdles may require both changes in the interaction between key actors and changes in legislation. Suggestions for change have been made by Austin et al. (2021) including, amongst others, improved education, financial and regulatory incentives that create viable business cases, and reimbursement strategies for off-label use. We propose the following recommendations for changes in legislation that build on these suggestions:

First, when reality shows that some repurposed drugs are not being registered and widespread off-label use is the result, supported by scientific evidence, other options to reach long-term availability and appropriate use driven by academia could be explored (“label change last” (Austin et al., 2021)). For example, this could entail close monitoring and structured assessment of off-label use by regulators and reimbursement authorities, and providing regulators with the right to change a label or add an indication to a label as proposed by Gyawali et al. (2021). Second, society should be willing to support rare disease drug repurposing by facilitating reimbursement at a fair price. When payers pressure for the lowest possible prices for generic drugs, sustainable commercial drug repurposing is not feasible. For example, a solution could be that governments compensate companies that repurpose drugs based on costs (Van den Berg et al., 2021), or that repurposed drugs are exempted from external reference pricing policies.

Next to changes in legislation, we propose two recommendations for improved interaction between key actors:

First, healthcare professionals involved in drug repurposing should become better educated in the regulatory field and understand the advantages of a marketing authorization over off-label use. Increasing knowledge in academia about the regulatory system, perhaps centralized on a national level as well as international efforts such as the STARS initiative can increase alignment (Starokozhko et al., 2021). Second, healthcare professionals involved in drug repurposing together with private and regulatory actors will have to learn and understand each other’s drive and language. Early dialogue between healthcare professionals involved in drug repurposing, industry, payers and regulators, will help to create a common ground and clear route to long-term availability and appropriate use of a drug. Also, involvement of academia may lead to public-private partnerships in which societal values are captured, limiting the possibilities for exploitation of monopolies.

## Conclusion

This study shows that drug repurposing for rare diseases mainly starts in academia, but there are many hurdles for these repurposed drugs to successfully reach patients. The results of our study may be used to operationalize the role of academic researchers and not-for-profit stakeholders in drug repurposing as highlighted by the European Commission (European Commission, 2020). We proposed changes in legislation or reimbursement schemes to ensure sustainable commercial drug repurposing. Yet, also the needs and skills of healthcare professionals involved in drug repurposing, industry and regulators need to become better aligned to stimulate successful marketing and reimbursement of repurposed drugs for patients with a rare disease.

## Data Availability

The original contributions presented in the study are included in the article/Supplementary Material, further inquiries can be directed to the corresponding author.

## References

[B1] Aceves S. S. Bastian J. F. Newbury R. O. Dohil R. (2007). Oral Viscous Budesonide: A Potential New Therapy for Eosinophilic Esophagitis in Children. Am. J. Gastroenterol. 102 (10), 2271–2280. 10.1111/j.1572-0241.2007.01379.x 17581266

[B2] Austin C. P. Mount B. A. Colvis C. M. (2021). Envisioning an Actionable Research Agenda to Facilitate Repurposing of Off-Patent Drugs. Nat. Rev. Drug Discov. 20, 723–724. 10.1038/d41573-021-00090-y 34045734

[B3] Bartstra J. W. de Jong P. A. Kranenburg G. Wolterink J. M. Isgum I. Wijsman A. (2020). Etidronate Halts Systemic Arterial Calcification in Pseudoxanthoma Elasticum. Atherosclerosis 292, 37–41. 10.1016/j.atherosclerosis.2019.10.004 31756632

[B4] BenEzra D. Pe'er J. Brodsky M. Cohen E. (1986). Cyclosporine Eyedrops for the Treatment of Severe Vernal Keratoconjunctivitis. Am. J. Ophthalmol. 101 (3), 278–282. 10.1016/0002-9394(86)90819-6 3953723

[B5] Bozdag S. Gumus K. Gumus O. Ünlü N. (2008). Formulation and *In Vitro* Evaluation of Cysteamine Hydrochloride Viscous Solutions for the Treatment of Corneal Cystinosis. Eur. J. Pharmaceutics Biopharmaceutics 70 (1), 260–269. 10.1016/j.ejpb.2008.04.010 18590953

[B6] Breckenridge A. Jacob R. (2018). Overcoming the Legal and Regulatory Barriers to Drug Repurposing. Nat. Rev. Drug Discov. 18 (1), 1–2. 10.1038/nrd.2018.92 29880920

[B7] Caban A. Pisarczyk K. Kopacz K. Kapuśniak A. Toumi M. Rémuzat C. (2017). Filling the gap in CNS Drug Development: Evaluation of the Role of Drug Repurposing. J. Mark Access Health Pol. 5 (1), 1299833. 10.1080/20016689.2017.1299833 PMC5405562 28473889

[B8] Crowther D. Bateman C. J. Vartan C. P. Whitehouse J. M. Malpas J. S. Fairley G. H. (1970). Combination Chemotherapy Using L-Asparaginase, Daunorubicin, and Cytosine Arabinoside in Adults with Acute Myelogenous Leukaemia. Br. Med. J. 4, 513–517. 10.1136/bmj.4.5734.513 4921703 PMC1820017

[B9] Davies E. H. Fulton E. Brook D. Hughes D. A. (2017). Affordable Orphan Drugs: a Role for Not-for-profit Organizations. Br. J. Clin. Pharmacol. 83 (7), 1595–1601. 10.1111/bcp.13240 28109021 PMC5465340

[B10] Dooms M. Cassiman D. Simoens S. (2016). Off-label Use of Orphan Medicinal Products: a Belgian Qualitative Study. Orphanet J. Rare Dis. 11 (1), 144–149. 10.1186/s13023-016-0507-y 27793155 PMC5086036

[B11] Dufier J. L. Dhermy P. Gubler M. C. Gagnadoux M. F. Broyer M. (1987). Ocular Changes in Long-Term Evolution of Infantile Cystinosis. Ophthalmic Paediatr. Genet. 8 (2), 131–137. 10.3109/13816818709028529 3658340

[B12] EMA (2020). Download Table of All EPARs for Human and Veterinary Medicines. Available at: https://www.ema.europa.eu/sites/default/files/Medicines_output_european_public_assessment_reports.xlsx (Accessed December 16, 2020).

[B13] European Commission (2021). Initiative Details. Available at: https://ec.europa.eu/info/law/better-regulation/have-your-say/initiatives/12767-Medicines-for-children-&-rare-diseases-updated-rules_en (Accessed July 5, 2021).

[B14] European Commission (2020). Pharmaceutical Strategy for Europe. Communiocation from the Commission to the European Parliament, the Council, the European Economic and Social Committee and the Committee of the Regions. Available at: https://eur-lex.europa.eu/legal-content/EN/TXT/?uri=CELEX:52020DC0761 (Accessed June 17, 2021).

[B15] Forslöw U. Geborek A. Hjelte L. Petrini B. Heurlin N. (2003). Early Chemotherapy for Non-tuberculous Mycobacterial Infections in Patients with Cystic Fibrosis. Acta Paediatr. 92 (8), 910–915. 10.1080/08035250310004388 12948065

[B16] Gyawali B. Darrow J. J. Kesselheim A. S. (2021). Integrating New Effectiveness Data into US Food and Drug Administration-Approved Drug Labeling. JAMA Intern. Med. 181 (7), 897–898. 10.1001/jamainternmed.2021.1994 33999146

[B17] Holman C. M. (2019). Momenta Pharm., Inc. V. Bristol-Myers Squibb Co. United States Court of Appeals of the Federal Circuit, 2019 915 F.3d 764. Biotechnol. L. Rep. 38 (2), 131–134. 10.1089/blr.2019.29113.cmh

[B18] Izquierdo I. Orsingher O. A. Berardi A. C. (1973). Effect of Cannabidiol and of Other Cannabis Sativa Compounds on Hippocampal Seizure Discharges. Psychopharmacologia 28 (1), 95–102. 10.1007/BF00413961 4714680

[B19] Kort E. Jovinge S. (2021). Drug Repurposing: Claiming the Full Benefit from Drug Development. Curr. Cardiol. Rep. 23 (6), 1–7. 10.1007/s11886-021-01484-5 33961142

[B20] Kranenburg G. de Jong P. A. Bartstra J. W. Lagerweij S. J. Lam M. G. Ossewaarde-van Norel J. (2018). Etidronate for Prevention of Ectopic Mineralization in Patients with Pseudoxanthoma Elasticum. J. Am. Coll. Cardiol. 71 (10), 1117–1126. 10.1016/j.jacc.2017.12.062 29519353

[B21] Kuehn H. S. Ouyang W. Lo B. Deenick E. K. Niemela J. E. Avery D. T. (2014). Immune Dysregulation in Human Subjects with Heterozygous Germline Mutations in CTLA4. Science 345 (6204), 1623–1627. 10.1126/science.1255904 25213377 PMC4371526

[B22] Langedijk J. Mantel-Teeuwisse A. K. Slijkerman D. S. Schutjens M. H. (2015). Drug Repositioning and Repurposing: Terminology and Definitions in Literature. Drug Discov. Today 20 (8), 1027–1034. 10.1016/j.drudis.2015.05.001 25975957

[B23] Langedijk J. Slijkerman D. S. Leufkens H. G. M. Schutjens M. D. B. Mantel-Teeuwisse A. K. (2016). “Continuous Innovation in the Drug Life Cycle,”. PhD thesis (Utrecht: Utrecht University). Challenges in innovations with well-known drugs: a quantitative and qualitative analysis. Appears in: Langedijk, J (2016)Available at: https://dspace.library.uu.nl/bitstream/handle/1874/342335/Langedijk.pdf?sequence=1 .

[B24] Lanz A. L. Riester M. Peters P. Schwerd T. Lurz E. Hajji M. S. (2021). Abatacept for Treatment-Refractory Pediatric CTLA4-Haploinsufficiency. Clin. Immunol. 229, 108779. 10.1016/j.clim.2021.108779 34116213

[B25] Lapides D. A. McDonald M. M. (2020). Inflammatory Manifestations of Systemic Diseases in the Central Nervous System. Curr. Treat. Options. Neurol. 22 (9), 26. 10.1007/s11940-020-00636-2 32834714 PMC7387810

[B26] Lee S. Moon J. S. Lee C. R. Kim H. E. Baek S. M. Hwang S. (2016). Abatacept Alleviates Severe Autoimmune Symptoms in a Patient Carrying a De Novo Variant in CTLA-4. J. Allergy Clin. Immunol. 137 (1), 327–330. 10.1016/j.jaci.2015.08.036 26478010

[B27] Lessin S. R. Duvic M. Guitart J. Pandya A. G. Strober B. E. Olsen E. A. (2013). Topical Chemotherapy in Cutaneous T-Cell Lymphoma: Positive Results of a Randomized, Controlled, Multicenter Trial Testing the Efficacy and Safety of a Novel Mechlorethamine, 0.02%, Gel in Mycosis Fungoides. JAMA Dermatol. 149 (1), 25–32. 10.1001/2013.jamadermatol.541 23069814 PMC3662469

[B28] Ministerie van Volksgezondheid Welzijn en Sport (2018). Antwoord op vragen van het lid Kooiman over het bericht dat medicatie voor botontkalking van de markt is gehaald terwijl het PXE-patiënten nieuwe hoop geeft. Available at: https://zoek.officielebekendmakingen.nl/ah-tk-20172018-1626.html (Accessed August 31, 2021).

[B29] National Institute for Care and Health Excellence (2021). Project Documents | Mexiletine for Treating Myotonia in Adults with Non-dystrophic Myotonic Disorders ID1488. Available at: https://www.nice.org.uk/guidance/indevelopment/gid-ta10432/documents (Accessed July 25, 2021). 40811590

[B30] Orphanet (2021). Orphanet: Pseudoxanthoma Elasticum. Available at: https://www.orpha.net/consor/cgi-bin/OC_Exp.php?lng=EN&Expert=758 (Accessed July 25, 2021).

[B31] Polamreddy P. Gattu N. (2019). The Drug Repurposing Landscape from 2012 to 2017: Evolution, Challenges, and Possible Solutions. Drug Discov. Today 24 (3), 789–795. 10.1016/j.drudis.2018.11.022 30513339

[B32] Postema P. G. Schwartz P. J. Arbelo E. Bannenberg W. J. Behr E. R. Belhassen B. (2020). Continued Misuse of Orphan Drug Legislation: a Life-Threatening Risk for Mexiletine. Eur. Heart J. 41 (5), 614–617. 10.1093/eurheartj/ehaa041 32006435

[B33] Pouget J. Serratrice G. (1983). Myotonia with Muscular Weakness Corrected by Exercise. The Therapeutic Effect of Mexiletine. Rev. Neurol. (Paris) 139 (11), 665–672. 6677978

[B34] Pushpakom S. Iorio F. Eyers P. A. Escott K. J. Hopper S. Wells A. (2018). Drug Repurposing: Progress, Challenges and Recommendations. Nat. Rev. Drug Discov. 18 (1), 41–58. 10.1038/nrd.2018.168 30310233

[B35] Risseeuw S. Bartstra J. Ossewaarde-van Norel J. Geurts L. J. Li C. H. Z. Imhof S. M. (2020). Is Arterial Stiffness in the Carotid Artery Associated with Choroidal Thinning in Patients with Pseudoxanthoma Elasticum or Controls. Acta Ophthalmol. 98, 492–499. 10.1111/aos.14346 31943777

[B36] Sagen J. V. Raeder H. Hathout E. Shehadeh N. Gudmundsson K. Baevre H. (2004). Permanent Neonatal Diabetes Due to Mutations in KCNJ11 Encoding Kir6.2: Patient Characteristics and Initial Response to Sulfonylurea Therapy. Diabetes 53, 2713–2718. 10.2337/diabetes.53.10.2713 15448106

[B37] Sakata Y. Shimada Y. Yoshino M. Kambe M. Futatsuki K. Nakao I. (1994). A Late Phase II Study of CPT-11, Irinotecan Hydrochloride, in Patients with Advanced Pancreatic Cancer. CPT-11 Study Group on Gastrointestinal Cancer. Gan To Kagaku Ryoho 21 (7), 1039–1046. 8210255

[B38] Salen G. Tint G. S. Eliav B. Deering N. Mosbach E. H. (1974). Increased Formation of Ursodeoxycholic Acid in Patients Treated with Chenodeoxycholic Acid. J. Clin. Invest. 53 (2), 612–621. 10.1172/JCI107596 11344576 PMC301505

[B39] Sardana D. Zhu C. Zhang M. Gudivada R. C. Yang L. Jegga A. G. (2011). Drug Repositioning for Orphan Diseases. Brief Bioinform 12 (4), 346–356. 10.1093/bib/bbr021 21504985

[B40] Schmidt-Hieber M. Blau I. W. Trenschel R. Andreesen R. Stuhler G. Einsele H. (2007). Reduced-toxicity Conditioning with Fludarabine and Treosulfan Prior to Allogeneic Stem Cell Transplantation in Multiple Myeloma. Bone Marrow Transpl. 39 (7), 389–396. 10.1038/sj.bmt.1705605 17310135

[B41] Seidenschnur K. E. K. Dressler C. Weller K. Nast A. Werner R. N. (2017). Off-label Prescriptions and Decisions on Reimbursement Requests in Germany - a Retrospective Analysis. J. Dtsch Dermatol. Ges 15 (11), 1103–1109. 10.1111/ddg.13345 29064628

[B42] Sheldon T. (2018). Dutch Hospital Makes Own Drug for Rare Condition after Manufacturer Hikes price to €170 000. BMJ 361, k2103. 10.1136/bmj.k2103 29748288

[B43] Shields C. L. Say E. A. Mashayekhi A. Garg S. J. Dunn J. P. Shields J. A. (2016). Assessment of CTLA-4 Deficiency-Related Autoimmune Choroidopathy Response to Abatacept. JAMA Ophthalmol. 134 (7), 844–846. 10.1001/jamaophthalmol.2016.1013 27258812

[B44] Siggs O. M. Russell A. Singh-Grewal D. Wong M. Chan P. Craig M. E. (2019). Preponderance of CTLA4 Variation Associated with Autosomal Dominant Immune Dysregulation in the MYPPPY Motif. Front. Immunol. 10, 1544–1546. 10.3389/fimmu.2019.01544 31396201 PMC6664875

[B45] Skoro-Sajer N. Bonderman D. Wiesbauer F. Harja E. Jakowitsch J. Klepetko W. (2007). Treprostinil for Severe Inoperable Chronic Thromboembolic Pulmonary Hypertension. J. Thromb. Haemost. 5 (3), 483–489. 10.1111/j.1538-7836.2007.02394.x 17319903

[B46] Starokozhko V. Kallio M. Kumlin Howell Å. Mäkinen Salmi A. Andrew-Nielsen G. Goldammer M. (2021). Strengthening Regulatory Science in Academia: STARS, an EU Initiative to Bridge the Translational gap. Drug Discov. Today 26 (2), 283–288. 10.1016/j.drudis.2020.10.017 33127567

[B47] Stunnenberg B. C. LoRusso S. Arnold W. D. Barohn R. J. Cannon S. C. Fontaine B. (2020). Guidelines on Clinical Presentation and Management of Nondystrophic Myotonias. Muscle Nerve 62, 430–444. 10.1002/mus.26887 32270509 PMC8117169

[B48] Tambuyzer E. Vandendriessche B. Austin C. P. Brooks P. J. Larsson K. Miller Needleman K. I. (2020). Therapies for Rare Diseases: Therapeutic Modalities, Progress and Challenges Ahead. Nat. Rev. Drug Discov. 19 (2), 93–111. 10.1038/s41573-019-0049-9 31836861

[B49] Tardi P. Johnstone S. Harasym N. Xie S. Harasym T. Zisman N. (2009). *In Vivo* maintenance of Synergistic Cytarabine:daunorubicin Ratios Greatly Enhances Therapeutic Efficacy. Leuk. Res. 33 (1), 129–139. 10.1016/j.leukres.2008.06.028 18676016

[B50] Technopolis and Ecorys (2020). Study to Support the Evaluation of the EU Orphan Regulation. Available at: https://ec.europa.eu/health/sites/default/files/files/paediatrics/docs/orphan-regulation_study_final-report_en.pdf (Accessed September 8, 2021).

[B51] Trip J. Drost G. G. van Engelen B. G. Faber C. G. (2006). Drug Treatment for Myotonia. Cochrane Database Syst. Rev., CD004762. 10.1002/14651858.cd004762.pub2 16437496 PMC9036524

[B52] van den Berg S. van der Wel V. de Visser S. J. Stunnenberg B. C. Timmers L. van der Ree M. H. (2021). Cost-Based Price Calculation of Mexiletine for Nondystrophic Myotonia. Value Health 24 (7), 925–929. 10.1016/j.jval.2021.02.004 34243835

[B53] Van Leeuwen E. M. Cuadrado E. Gerrits A. M. Witteveen E. de Bree G. J. (2018). Treatment of Intracerebral Lesions with Abatacept in a CTLA4-Haploinsufficient Patient. J. Clin. Immunol. 38 (4), 464–467. 10.1007/s10875-018-0511-1 29796761

[B54] Verbaanderd C. Rooman I. Huys I. (2021). Exploring New Uses for Existing Drugs: Innovative Mechanisms to Fund Independent Clinical Research. Trials 22 (1), 322. 10.1186/s13063-021-05273-x 33947441 PMC8093905

[B55] Verbaanderd C. Rooman I. Meheus L. Huys I. (2020). On-Label or Off-Label? Overcoming Regulatory and Financial Barriers to Bring Repurposed Medicines to Cancer Patients. Front. Pharmacol. 10, 1664. 10.3389/fphar.2019.01664 32076405 PMC7006723

[B56] Volkskrant (2019). Nijmeegse onderzoekers bewijzen nieuwe toepassing oud medicijn – en dus verzestienvoudigt farmaceut de vraagprijs. Available at: https://www.volkskrant.nl/wetenschap/nijmeegse-onderzoekers-bewijzen-nieuwe-toepassing-oud-medicijn-en-dus-verzestienvoudigt-farmaceut-de-vraagprijs∼bae26adb/ (Accessed September 8, 2021).

[B57] Volkskrant (2018). Wat als medicatie die in Nederland op papier niet meer bestaat toch uitkomst biedt. Available at: https://www.volkskrant.nl/wetenschap/wat-als-medicatie-die-in-nederland-op-papier-niet-meer-bestaat-toch-uitkomst-biedt∼b5d13ea1/? (Accessed September 8, 2021).

[B58] Wagener D. J. Verdonk H. E. Dirix L. Y. Catimel G. Siegenthaler P. Buitenhuis M. (1995). Phase II Trial of CPT-11 in Patients with Advanced Pancreatic Cancer, an EORTC Early Clinical Trials Group Study. Ann. Oncol. 6 (2), 129–132. 10.1093/oxfordjournals.annonc.a059107 7786820

[B59] Weers J. Metzheiser B. Taylor G. Warren S. Meers P. Perkins W. R. (2009). A Gamma Scintigraphy Study to Investigate Lung Deposition and Clearance of Inhaled Amikacin-Loaded Liposomes in Healthy Male Volunteers. J. Aerosol Med. Pulm. Drug Deliv. 22 (2), 131–138. 10.1089/jamp.2008.0693 19422313

[B60] WHO Collaborating Centre for Drug Statistics Methodology (2021). WHOCC - Structure and Principles. Available at: https://www.whocc.no/atc/structure_and_principles/ (Accessed August 30, 2021).

[B61] Wiesner A. Szuta M. Galanty A. Paśko P. (2021). Optimal Dosing Regimen of Osteoporosis Drugs in Relation to Food Intake as the Key for the Enhancement of the Treatment Effectiveness-A Concise Literature Review. Foods 10 (4). 10.3390/foods10040720 PMC8067335 33805435

[B62] Zorginstituut Nederland (2021b). GVS Advice Mexiletine (Namuscla®) for the Treatment of Non-dystrophic Myotonia. Available at: https://www.zorginstituutnederland.nl/publicaties/adviezen/2021/01/14/gvs-advies-mexiletine-namuscla-bij-de-behandeling-van-non-dystrofische-myotonie (Accessed July 25, 2021).

[B63] Zorginstituut Nederland (2021a). ORENCIA INFPDR FLACON 250MG. Available at: https://www.medicijnkosten.nl/medicijn?artikel=ORENCIA+INFPDR+FLACON+250MG&id=28efdda9ec746fe2d8501f284f3476bf (Accessed August 30, 2021).

[B64] Zung A. Glaser B. Nimri R. Zadik Z. (2004). Glibenclamide Treatment in Permanent Neonatal Diabetes Mellitus Due to an Activating Mutation in Kir6.2. J. Clin. Endocrinol. Metab. 89 (11), 5504–5507. 10.1210/jc.2004-1241 15531505

